# Mumps Vaccine-Associated Aseptic Meningitis Complicated by Syndrome of Inappropriate Antidiuretic Hormone Secretion

**DOI:** 10.7759/cureus.84656

**Published:** 2025-05-23

**Authors:** Maho Yuki, Tatsuya Kinoshita, Saori Yamada, Taku Tsuchida, Yosuke Hara

**Affiliations:** 1 Pediatrics, Ina Central Hospital, Ina, JPN

**Keywords:** aseptic meningitis, hyponatremia, mumps vaccine, pediatrics, syndrome of inappropriate antidiuretic hormone secretion (siadh), vaccine-associated adverse events

## Abstract

Mumps vaccine is generally safe; however, aseptic meningitis remains a rare but recognized adverse event. We report the case of a 15-year-old male who developed a progressive headache and hyponatremia, leading to a diagnosis of syndrome of inappropriate antidiuretic hormone secretion (SIADH). As his symptoms worsened, CSF analysis revealed pleocytosis with monocyte predominance and elevated protein levels, resulting in a diagnosis of aseptic meningitis. A detailed history showed that he had received a mumps vaccine 27 days prior to admission. PCR testing of the CSF detected mumps virus, and direct sequencing confirmed 100% identity with the Torii vaccine strain. Although the incidence of vaccine-associated aseptic meningitis is significantly lower than that following natural mumps infection, clinicians should remain vigilant, particularly in patients presenting with neurological symptoms and SIADH after mumps vaccination. Early recognition of CNS involvement and careful review of vaccination history are essential for accurate diagnosis and appropriate management. This case underscores the importance of considering vaccine-associated aseptic meningitis in the differential diagnosis of patients with hyponatremia and progressive headache following mumps vaccination, even when vaccination occurs beyond the routine immunization age.

## Introduction

Mumps is an acute viral infection that primarily affects children and can lead to complications such as aseptic meningitis, orchitis, and sensorineural hearing loss [1]. The introduction of vaccination programs has significantly reduced the global incidence of these complications. In Japan, the measles-mumps-rubella (MMR) vaccine was introduced as part of the routine immunization schedule in 1989 [2]. However, due to reports of vaccine-associated aseptic meningitis, the MMR vaccination program was discontinued in 1993 [2,3]. Since then, mumps vaccination has been offered on a voluntary basis using monovalent vaccines derived from either the Torii or Hoshino strains [4,5]. These live attenuated strains were developed domestically in Japan. The Torii strain, introduced in 1971 by Torii Pharmaceutical Co., Ltd., is known for its clinical efficacy and stable production profile. The Hoshino strain, developed in 1974 by Professor Hoshino and colleagues at Osaka University, was later adopted due to its favorable safety profile. Compared to older strains such as Urabe, both the Torii and Hoshino strains exhibit reduced neurovirulence. Although the incidence of vaccine-associated aseptic meningitis is substantially lower (approximately 0.03-0.16%) than that of natural mumps infection (approximately 1.24%) [4,5], such adverse events still occur. Here, we report a rare case of mumps vaccine-associated aseptic meningitis complicated by syndrome of inappropriate antidiuretic hormone secretion (SIADH), which developed approximately one month after vaccination. This case is particularly noteworthy as the patient received the vaccine at 15 years of age, beyond the typical recommended age for mumps immunization in Japan.

## Case presentation

A 15-year-old male presented with high fever, nausea, and headache and was referred to our hospital after two days of antipyretic treatment by a local physician. On admission, his body temperature was 39.5°C, and he reported a mild headache; however, he showed no disturbance of consciousness or hypertension (blood pressure: 129/55 mmHg). No meningeal signs, ophthalmologic symptoms, or other physical abnormalities were observed. Laboratory findings revealed mild leukocytosis and hyponatremia but no inflammatory response (Table 1). Multiplex PCR testing did not detect any infectious pathogens.

**Table 1:** Clinical laboratory findings on admission Abnormal values are shown in bold.

Test	Observed value	Reference range
White blood cell	8.76 × 10³/μL	3.30-8.60 × 10³/μL
Neutrophils	73.30%	NA
Lymphocytes	16.20%	NA
Hemoglobin	16.4 g/dL	13.7-16.8 g/dL
Platelets	173 × 10³/μL	158-348 × 10³/μL
Blood urea nitrogen	19.1 mg/dL	8.0-20.0 mg/dL
Serum creatinine	0.86 mg/dL	0.65-1.07 mg/dL
Sodium	128 mmol/L	138-145 mmol/L
Potassium	4.1 mmol/L	3.6-4.8 mmol/L
Chloride	93 mmol/L	101-108 mmol/L
C-reactive protein	0.01 mg/dL	0.00-0.14 mg/dL

After admission, his hyponatremia worsened, accompanied by a decrease in plasma osmolality, which led to a diagnosis of SIADH (Table 2). Hypothyroidism was also observed.

**Table 2:** Clinical laboratory findings on the day after admission Abnormal values are shown in bold. ACTH, adrenocorticotropic hormone; FENa, fractional excretion of sodium; FT3, free triiodothyronine; FT4, free thyroxine; TSH, thyroid-stimulating hormone

Test	Observed value	Reference range
White blood cell	8.18 × 10³/μL	3.30-8.60 × 10³/μL
Hemoglobin	15.3 g/dL	13.7-16.8 g/dL
Platelets	169 × 10³/μL	158-348 × 10³/μL
Sodium	126 mmol/L	138-145 mmol/L
Potassium	3.8 mmol/L	3.6-4.8 mmol/L
Chloride	93 mmol/L	101-108 mmol/L
C-reactive protein	0.01 mg/dL	0.00-0.14 mg/dL
Serum osmolality	272 mOsm/kg	275-290 mOsm/kg
TSH	0.11 mIU/L	0.61-4.23 mIU/L
FT3	1.67 pg/mL	2.30-4.00 pg/mL
FT4	1.28 ng/dL	0.90-1.70 ng/dL
Cortisol	11.1 μg/dL	4.0-18.3 μg/dL
ACTH	9.7 pg/mL	7.2-63.3 pg/mL
Vasopressin	1.1 pg/mL	<2.8 pg/mL
Urine osmolality	633 mOsm/kg	50-1300 mOsm/kg
FENa	1.14%	NA

**Figure 1:**
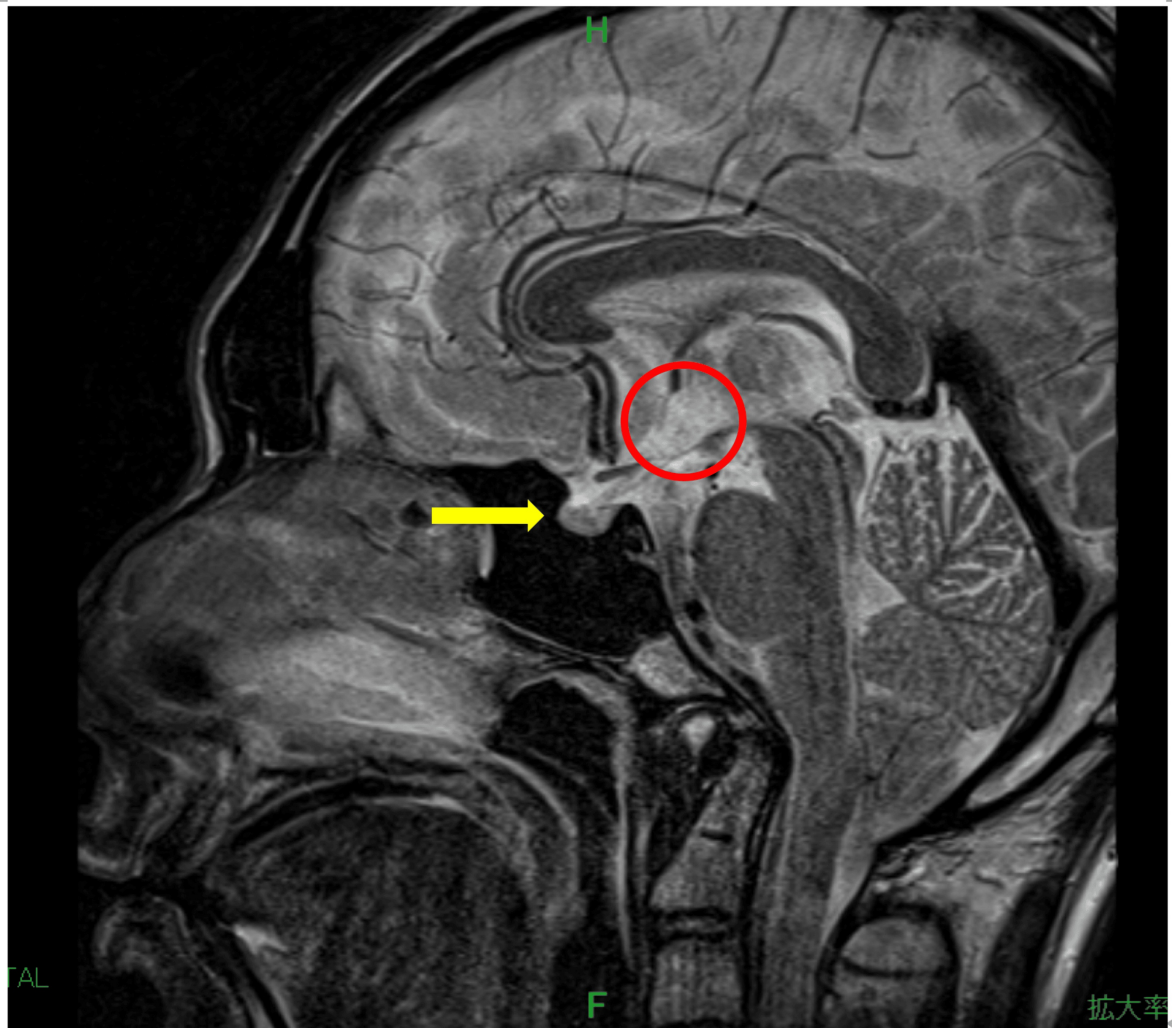
Sagittal T2-weighted MRI of the hypothalamus and pituitary gland on hospital day 1 The hypothalamus is indicated by a red circle, and the pituitary gland is marked with a yellow arrowhead.

**Figure 2:**
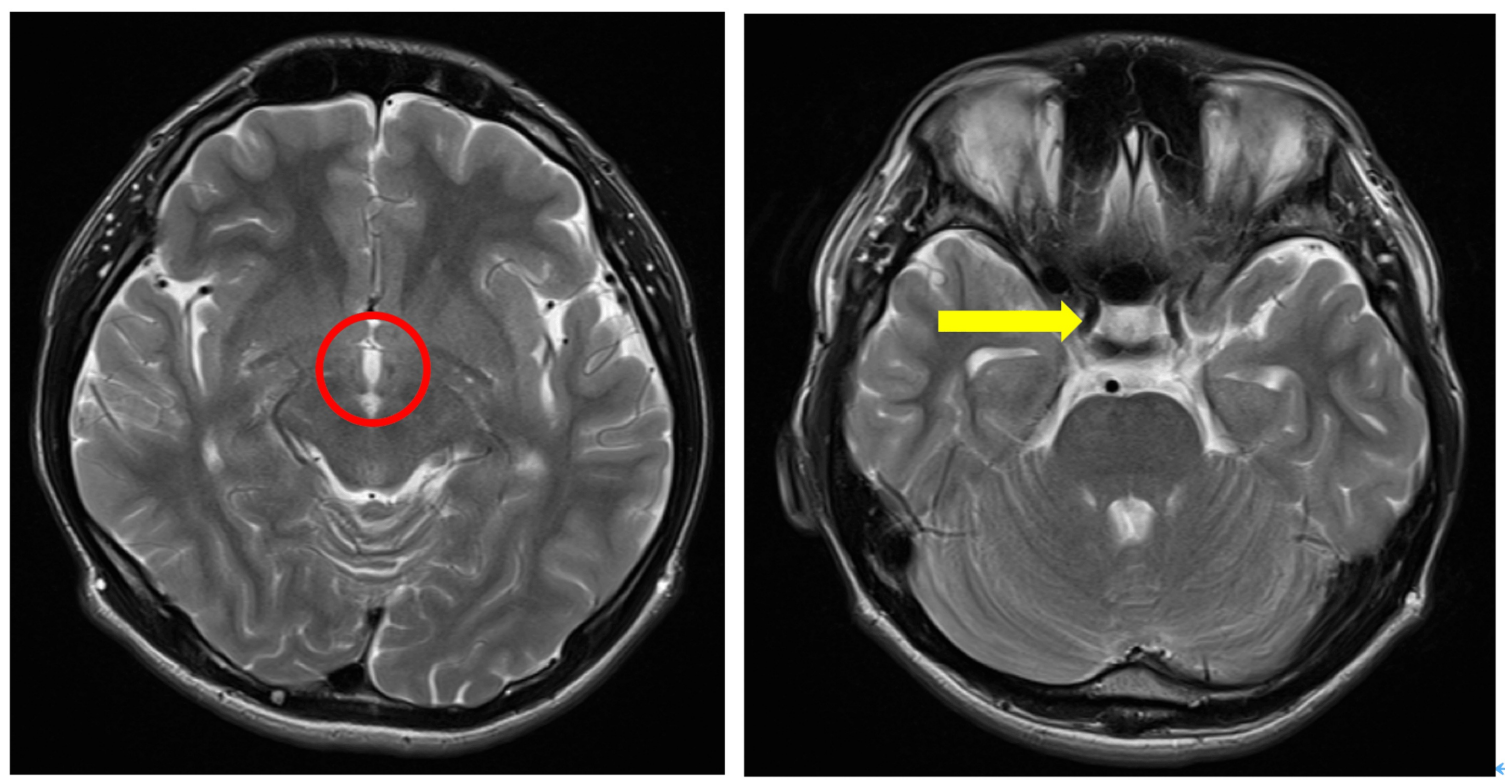
Axial T2-weighted MRI of the hypothalamus and pituitary gland on hospital day 1 The hypothalamus is indicated by a red circle, and the pituitary gland is marked with a yellow arrowhead.

However, brain MRI revealed no abnormalities in the hypothalamus or pituitary gland (Figure 1, Figure 2).

Although the headache was initially tolerable upon admission, it gradually worsened. By hospital day 2, the patient experienced a persistent, severe headache that impaired his ability to walk. On hospital day 3, a lumbar puncture was performed, which revealed pleocytosis with monocyte predominance and elevated protein levels in the CSF, leading to a diagnosis of aseptic meningitis (Table 3).

**Table 3:** CSF analysis on hospital day 3 Abnormal values are shown in bold.

Test	Observed value	Reference range
Turbidity	Positive	Negative
Cell count	317/μL	<5/μL
Mononuclear cells	99%	NA
Polymorphonuclear	1%	NA
CSF protein	67.5 mg/dL	10-40 mg/dL
CSF glucose	49 mg/dL	40-75 mg/dL

**Figure 3:**
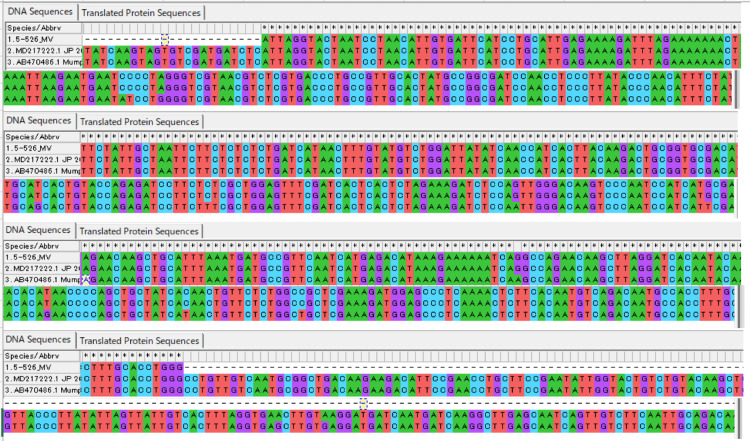
Direct sequencing analysis RT-PCR targeting the SH region of the mumps virus yielded a positive result. Sequencing was performed using the forward primer SHnes1 and the reverse primer SH4, and the resulting nucleotide sequence was analyzed. 5-526_MV: nucleotide sequence of the mumps virus detected in the patient’s CSF; MD217222.1 JP: mumps virus vaccine strain Torii; AB470486.1: mumps virus vaccine strain Hoshino A 100% sequence identity was confirmed between the patient-derived virus and the Torii vaccine strain.

With fluid restriction and oral administration of tolvaptan, both symptoms and laboratory findings gradually improved, and by hospital day 8, the serum sodium level had normalized to 138 mmol/L. A detailed history revealed that the patient had received a mumps vaccine 27 days prior to admission. There was no evidence of a regional outbreak or close contact related to the mumps virus. Subsequent PCR testing of the CSF detected the presence of the mumps virus. Further direct sequencing analysis confirmed 100% sequence identity with the Torii strain used in the vaccine (Figure 3).

After discharge, the patient remained free of any recurrence of neurological symptoms. Although thyroid-stimulating hormone levels remained low, free triiodothyronine and free thyroxine levels were within normal ranges. To further assess thyroid function, a thyrotropin-releasing hormone stimulation test was performed, which demonstrated a normal response.

## Discussion

Aseptic meningitis is a known but rare adverse reaction following mumps vaccination. In this case, a 15-year-old male developed aseptic meningitis approximately one month after receiving a mumps vaccine. Mumps virus was detected in the CSF, and direct sequencing confirmed 100% sequence identity with the Torii vaccine strain. There were no reported outbreaks of wild-type mumps in the region at that time, and the patient had no known close contact with infected individuals, making infection with a wild-type strain unlikely.

The most common adverse reaction to mumps vaccination is transient parotid gland swelling, typically occurring around three weeks post-vaccination and observed in approximately 2-3% of recipients. In contrast, aseptic meningitis is a more serious but much less frequent adverse event, usually occurring two to three weeks after vaccination. In the present case, onset occurred 27 days after vaccination, which is within an acceptable range of the expected time frame. In Japan, the use of the MMR vaccine was discontinued in 1993 due to concerns about vaccine-associated aseptic meningitis. Since then, only monovalent mumps vaccines derived from the Torii and Hoshino strains have been used. According to Nagai et al. (2007), the incidence of aseptic meningitis following monovalent mumps vaccination is estimated to be between 0.03% and 0.16%, which is significantly lower than the incidence associated with natural mumps infection (1.24%) [5].

In Japan, mumps vaccination is recommended at one to two years of age for the first dose and five to seven years for the second dose [4]. However, in this case, the patient received the vaccine at the age of 15, which is atypical. While most vaccine-associated adverse events are reported in younger children, this case underscores the importance of remaining vigilant for complications in older adolescents receiving catch-up immunizations. Clinicians should be aware that such events are not limited to the routine immunization age group. Delayed immunization may alter immune responses in older individuals, potentially increasing the risk of atypical or exaggerated reactions. Some reports also suggest that deviations from the recommended vaccination schedule are associated with an increased risk of severe infections and hospitalization [6]. These findings support the notion that adhering to the recommended immunization schedule not only optimizes immunogenicity but may also reduce the risk of adverse events, particularly in older recipients undergoing catch-up vaccination.

In this case, progressive headache and hyponatremia due to SIADH were key clinical clues that led to the diagnosis of aseptic meningitis. SIADH, characterized by water retention and hyponatremia resulting from excessive ADH secretion, is a known complication of CNS infections, including viral meningitis. The presumed mechanism involves inflammation or irritation of the hypothalamus or neurohypophysis, which may disrupt ADH regulation [7]. Although brain imaging revealed no structural abnormalities, functional disturbance caused by meningitis may have triggered SIADH. Clinicians should consider SIADH in the context of CNS infections, and recognizing atypical findings alongside a thorough vaccination history is critical for accurate diagnosis and effective management.

To evaluate the causal relationship between the mumps vaccine and the onset of aseptic meningitis in this case, we applied the World Health Organization-Uppsala Monitoring Centre system for standardized case causality assessment. The relationship was classified as “probable/likely,” as the adverse event occurred within a reasonable time frame, virological evidence supported vaccine involvement, and no alternative cause was identified. Although we did not apply the Brighton Collaboration Case Definition - given its specificity for surveillance data - the clinical and laboratory findings in this case are consistent with a vaccine-associated adverse event.

While this case offers valuable clinical insights, we acknowledge the limitations inherent to a single case report, particularly the inability to establish definitive causality. Further surveillance and larger studies are needed to better understand the incidence, risk factors, and pathophysiology of vaccine-associated aseptic meningitis.

## Conclusions

This case highlights the importance of considering mumps vaccine-associated aseptic meningitis in the differential diagnosis of patients presenting with hyponatremia, progressive headache, and recent mumps vaccination. Early recognition of SIADH and CNS involvement can facilitate timely diagnosis and appropriate management. Confirming vaccination history remains crucial for the accurate identification of vaccine-related complications, even outside the typical vaccination age range.
